# Clinical Associations of Preoperative and Postoperative Serum CEA and Lung Cancer Outcome

**DOI:** 10.3389/fmolb.2021.686313

**Published:** 2021-10-27

**Authors:** Zonglin Jiao, Shoubo Cao, Jianhua Li, Nan Hu, Yinghui Gong, Linduo Wang, Shi Jin

**Affiliations:** ^1^ Department of Medical Oncology, Harbin Medical University Cancer Hospital, Harbin, China; ^2^ Department of Medical and Radiation Oncology, Linyi People’s Hospital, Linyi, China; ^3^ Department of Neurosurgery, National Cancer Center/National Clinical Research Center for Cancer/Cancer Hospital and Shenzhen Hospital, Chinese Academy of Medical Sciences and Peking Union Medical College, Shenzhen, China; ^4^ Department of Oncology, Heilongjiang Agricultural Reclamation Bureau General Hospital, Harbin, China; ^5^ Department of Medical Oncology, National Cancer Center/National Clinical Research Center for Cancer/Cancer Hospital and Shenzhen Hospital, Chinese Academy of Medical Sciences and Peking Union Medical College, Shenzhen, China

**Keywords:** lung cancer, carcinoembryonic antigen, prognosis, stage I, stage II, stage III

## Abstract

**Background:** Serum carcinoembryonic antigen (CEA), a classic tumour marker, is widely used in lung cancer in clinical practice. Nevertheless, few studies have elucidated the influence of dynamic changes in CEA in the perioperative phases, as a prognostic indicator, on lung cancer prognosis.

**Methods:** This retrospective cohort analysis included consecutive patients with stage I-III lung cancer who underwent curative resection between December 2010 and December 2014. The patients were grouped into three cohorts: group A included patients with normal preoperative CEA, group B included patients with elevated preoperative CEA but normal postoperative CEA, and group C included patients with elevated preoperative and postoperative CEA. Five-year overall survival (OS) was estimated by Kaplan-Meier analysis (log-rank test). Multivariate analyses were performed with Cox proportional hazard regression.

**Results:** A total of 1662 patients with stage I-III lung cancer were enrolled in our study. Patients with normal preoperative CEA had 15.9 and 20.1% better 3- and 5-year OS rates than the cohort with elevated preoperative CEA (*p* < 0.001). Furthermore, group C had 36.0 and 26.6% lower 5-year OS rates (*n* = 74, 32.4%) than group A (*n* = 1188, 68.4%) and group B (*n* = 139, 59.0%) (*p* < 0.001). Group B had poorer OS than group A (*p* = 0.016). For patients with different pathological TNM stages, subgroup analyses showed that group C had the shortest OS in stages I and II (*p* < 0.05), and patients with a post-preoperative CEA increment had poorer OS than those without an increment (*p* = 0.029). Multivariate analyses suggested that group C (HR = 2.0, 95% CI, 1.5–2.7, *p* < 0.001) rather than the group with normalized postoperative CEA (HR = 1.2, 95% CI, 0.9–1.5, *p* = 0.270) was an independent prognostic factor. In subgroup analysis of adenocarcinoma (ADC), survival analyses suggested that group C predicted a worse prognosis. Multivariate analysis of ADC indicated that group C was an independent adverse prognostic factor (HR = 1.9, 95% CI, 1.4–2.7, *p* < 0.001).

**Conclusions:** Combined elevated preoperative and postoperative CEA is an independent adverse prognostic factor for stage I-III lung adenocarcinoma. Additionally, routine perioperative detection of serum CEA can yield valuable prognostic information for patients after lung cancer surgery.

## Introduction

Cancer Statistics, 2021 indicated that in both sexes, lung cancer is a commonly diagnosed cancer (11.4% of total cancer cases) and the leading cause of cancer mortality (18.0% of total cancer deaths) (Sung et al., 2021). There is no doubt that lung cancer will place a tremendous burden on society in the coming decades.

Serum tumour markers are widely used in the diagnosis and prognostic monitoring of lung cancer, and carcinoembryonic antigen (CEA) is one of the most sensitive markers (Moertel et al., 1993; Plebani et al., 1995; Molina et al., 2009). CEA is a glycoprotein associated with cell adhesion and is usually produced during foetal development but ceases to be secreted before birth. Specifically, CEA is a glycosylphosphatidylinositol (GPI) cell surface-anchored glycoprotein that serves as a ligand for L-selectin and E-selectin but is usually not present in healthy adult blood (Thomas et al., 2008; Konstantopoulos and Thomas, 2009). CEA has been widely acknowledged and recommended as a reliable tumour marker in colorectal cancer. However, it also plays vital roles in lung cancer diagnosis, progression, recurrence, metastasis, and various treatment effects (Wang et al., 2014; Chen et al., 2018; Konishi et al., 2018). Some studies have reported that a high preoperative serum CEA level is an independent prognostic factor and that a high postoperative level of serum CEA always indicates a poor prognosis in lung cancer (Sawabata et al., 2002; Sawabata et al., 2004a; Okada et al., 2004). For non-small lung cancer (NSCLC), in patients with a high preoperative serum CEA level, their postoperative serum CEA level has better prognostic value than their post/preoperative serum CEA ratio (Tomita et al., 2015). However, some studies have reported controversial findings on whether serum CEA can serve as a prognostic and predictive marker in lung cancer. The authors concluded that CEA is of little use as a diagnostic marker for small cell lung cancer (SCLC) and NSCLC (Ford et al., 1981; Schneider et al., 2000; Hatzakis et al., 2002).

The value of CEA in lung cancer prognosis related to dynamic changes in preoperative and postoperative serum CEA levels has not been demonstrated systematically. In our analysis, we attempted to observe whether perioperative changes in CEA could provide more prognostic information. More specifically, we sought to explore whether patients with an elevated preoperative CEA level that normalizes after curative resection have a similar risk of death as those with a CEA level that is elevated throughout the perioperative period.

## Methods

### Study Design and Data Collection

This study was a retrospective clinical study and was approved by the Ethics Committee of Harbin Medical University Cancer Hospital. All the consecutive patients were from the Harbin Medical University Cancer Hospital and pathologically diagnosed with stage I to III lung cancer between December 2010 and December 2014. The exclusion criteria were as follows: *1*) treatment for malignancy within 5 years; *2*) no available preoperative CEA value; *3*) preoperative chemotherapy or radiotherapy; and *4*) a lack of complete follow-up information.

Data on the patients’ clinical characteristics, including demographics, pathological reports, and perioperative clinical outcomes, were acquired from the departmental database and electronic patient records. Pathological stage was defined based on the seventh edition of the AJCC lung cancer criteria. We defined preoperative CEA as the CEA value recorded closest to the surgery time, and postoperative CEA as the CEA value recorded within 12 weeks after surgery but before postoperative adjuvant therapy. The normal reference CEA value for the assay was 0.0–5.0 ng/ml (Roche Cobas e 602 ECL analyser). Patients were grouped as follows: *1*) (Group A) normal preoperative CEA group, patients with a normal preoperative CEA level (≤5 ng/ml); *2*) (Group B) normalized postoperative CEA group, patients with an elevated preoperative CEA level (>5 ng/ml) but a normal postoperative CEA level; and *3*) (Group C) elevated postoperative CEA group, patients with elevated preoperative and postoperative CEA levels. We also used a CEA cut-off of 10.0 ng/ml for repeat analyses (Konishi et al., 2018). According to authoritative guidelines, all patients were followed up by history, physical examination, chest CT and PET/CT or MRI. Survival statuses were effectively updated by telephone, email, or medical history. Overall survival (OS) was defined as the time from surgery to death or last follow-up. Patients alive at the last follow-up date were censored.

### Statistical Analysis

All statistical data in this study were analysed with IBM SPSS 21.0 statistical software and GraphPad Prism 8.0. Three- and 5-year overall survival (OS) was estimated by the Kaplan-Meier method with the log-rank test for univariate analysis. The various indicators of prognosis with *p*-values of less than 0.05 in the univariate analysis were examined in multivariate analyses. Hazard ratios (HRs), 95% confidence intervals (CIs), and the effects of prognostic factors were estimated by Cox regression in the multivariate Cox regression. All *p*-values were two-sided, and *p*-values < 0.05 were considered significant.

## Results

**FIGURE 1:**
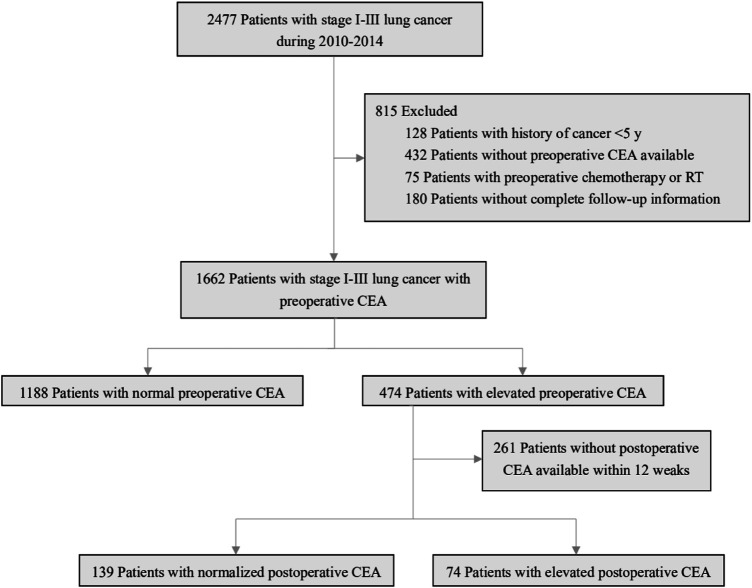
Study design.

A total of 2477 consecutive patients with stage I-III lung cancer who underwent curative resection were included in our study. According to the exclusion criteria, patients who had prior cancer treatment within 5 years (*n* = 128), were missing a preoperative CEA value (*n* = 432), had received preoperative chemotherapy or radiotherapy (*n* = 75), or had incomplete follow-up information (*n* = 180) were excluded. Among the remaining 1662 patients, 1188 (71.5%) had a normal preoperative CEA level, and 474 (28.5%) had an elevated CEA level. A total of 261 of the 474 patients with an elevated preoperative CEA level had no available postoperative CEA data within 12 weeks; 139 of the remaining patients had a normalized postoperative CEA value, and 74 had an elevated postoperative CEA value (Figure 1).

Descriptive statistics of patient and tumour characteristics for 1401 patients are shown in Table 1. There were 859 males (61.3%) and 542 females (38.7%) in our study. The median age (IQR) of all these patients was 58 (52–64) years (mean age, 57.9 ± 8.6 years). More than half of the patients (56.6%, *n* = 796) had a smoking history. Most patients (83.6%, *n* = 1171) did not have a family history of cancer. The main types of pathology included adenocarcinoma (ADC; 61.7%, *n* = 864), squamous cell carcinoma (SCC; 34.3%, *n* = 480) and others (4.1%, *n* = 57). For T stage, 648 patients (46.3%) had T1 disease, and 561 (40.0%) had T2 disease, while there were only 96 in stage T3 or T4. A total of 479 patients had lymph node metastasis, including 182 patients (13%) with N1 disease, 294 (21%) with N2 disease and 3 (0.2%) with N3 disease. The median (IQR) preoperative CEA level was 2.7 (1.8–4.1) ng/ml. In 608 patients with postoperative CEA data, the median postoperative CEA level was 2.1 (1.3–3.5) ng/ml, and the median (IQR) days from surgery to CEA testing was 38 (32.0–54.8) days (Table 2). The median (IQR) follow-up time was 76 (67–87) months. According to our follow-up data, a total of 537 patients died. The 5-year OS rate for all these patients was 65.6%.

**TABLE 1:** Patient and tumor characteristics.

Characteristic	All (*N* = 1401)	Normal preoperative CEA (*N* = 1188)	Normalized postoperative CEA (*N* = 139)	Elevated postoperative CEA (*N* = 74)
Gende, no. (%) of patients				
Male	859 (61.3)	748 (63.0)	74 (53.2)	37 (50.0)
Female	542 (38.7)	440 (37.0)	65 (46.8)	37 (50.0)
Age, years				
Median (IQR)	58 (52.0–64.0)	58 (52.3–64.0)	57 (51.0–64.0)	58 (51.8–63.0)
Mean (SD)	57.9 (8.6)	58.0 (8.5)	55.6 (9.4)	57.4 (7.8)
Smoking				
Yes	793 (56.6)	694 (58.4)	69 (49.6)	30 (40.5)
No	608 (43.4)	494 (41.6)	70 (50.4)	44 (59.5)
Family History				
Yes	230 (16.4)	205 (17.3)	13 (9.4)	12 (16.2)
No	1171 (83.6)	983 (82.7)	126 (90.6)	62 (83.8)
Pathology, no. (%) of patients				
ADC	864 (61.7)	696 (58.6)	102 (73.4)	66 (89.2)
SCC	480 (34.3)	454 (38.2)	23 (16.5)	3 (4.1)
Others	57 (4.1)	38 (3.2)	14 (10.1)	5 (6.8)
T Stage, no. (%) of patients				
T1	648 (46.3)	595 (50.1)	38 (27.3)	15 (20.3)
T2	561 (40.0)	438 (36.9)	79 (56.8)	44 (59.5)
T3	96 (6.9)	80 (6.7)	13 (9.4)	3 (4.1)
T4	96 (6.9)	75 (6.3)	9 (6.5)	12 (16.2)
N Stage, no. (%) of patients				
N0	922 (65.8)	837 (70.5)	64 (46)	21 (28.4)
N1	182 (13)	149 (12.5)	24 (17.3)	9 (12.2)
N2	294 (21)	199 (16.8)	51 (36.7)	44 (59.5)
N3	3 (0.2)	3 (0.3)	0 (0)	0 (0)
AJCC 7th ed. stage, no. (%) of patients				
IA1	66 (4.7)	66 (5.6)	0 (0)	0 (0)
IA2	235 (16.8)	224 (18.9)	9 (6.5)	2 (2.7)
IA3	190 (13.6)	178 (15.0)	8 (5.8)	4 (5.4)
IB	265 (18.9)	227 (19.1)	27 (19.4)	11 (14.9)
IIA	55 (3.9)	47 (4.0)	7 (5)	1 (1.4)
IIB	219 (15.6)	183 (15.3)	31 (22.3)	6 (8.1)
IIIA	310 (22.1)	221 (18.6)	49 (35.3)	40 (54.1)
IIIB	61 (4.4)	43 (3.6)	8 (5.8)	10 (13.5)
Preoperative CEA, ng/ml				
Median (IQR)	2.7 (1.8–4.1)	2.4 (1.6–3.4)	8.6 (6.2–15.1)	26.9 (9.6–77.5)
Mean (SD)	7.2 (33.3)	2.5 (1.1)	15.6 (29.2)	67.9 (124.0)
Adjuvant chemotherapy, no. (%) of patients				
Yes	560 (40.0)	402 (33.8)	109 (78.4)	49 (66.2)
No	802 (57.2)	755 (63.6)	25 (18)	22 (29.7)
Unknown	39 (2.8)	31 (2.6)	5 (3.6)	3 (4.1)

**TABLE 2:** Patient with postoperative CEA characteristics.

Characteristic	All (*N* = 608)	Normal preoperative CEA (*N* = 395)	Normalized postoperative CEA (*N* = 139)	Elevated postoperative CEA (*N* = 74)



Postoperative CEA, ng/ml					
Median (IQR)	2.1 (1.3–3.5)	1.67 (1.2–2.4)	2.6 (1.7–3.5)	11.1 (6.4–31.6)	
Mean (SD)	10.6 (63.5)	3.8 (15.7)	2.6 (1.1)	62.0 (170.6)	
Days from surgery to CEA testing					
Median (IQR)	38 (32.0–54.8)	38 (32.0–54.0)	37 (32.0–55.0)	39.5 (33.0–54.3)	
Mean (SD)	43.7 (16.1)	43.5 (16.4)	43.7 (15.6)	44.6 (16.0)	

**FIGURE 2:**
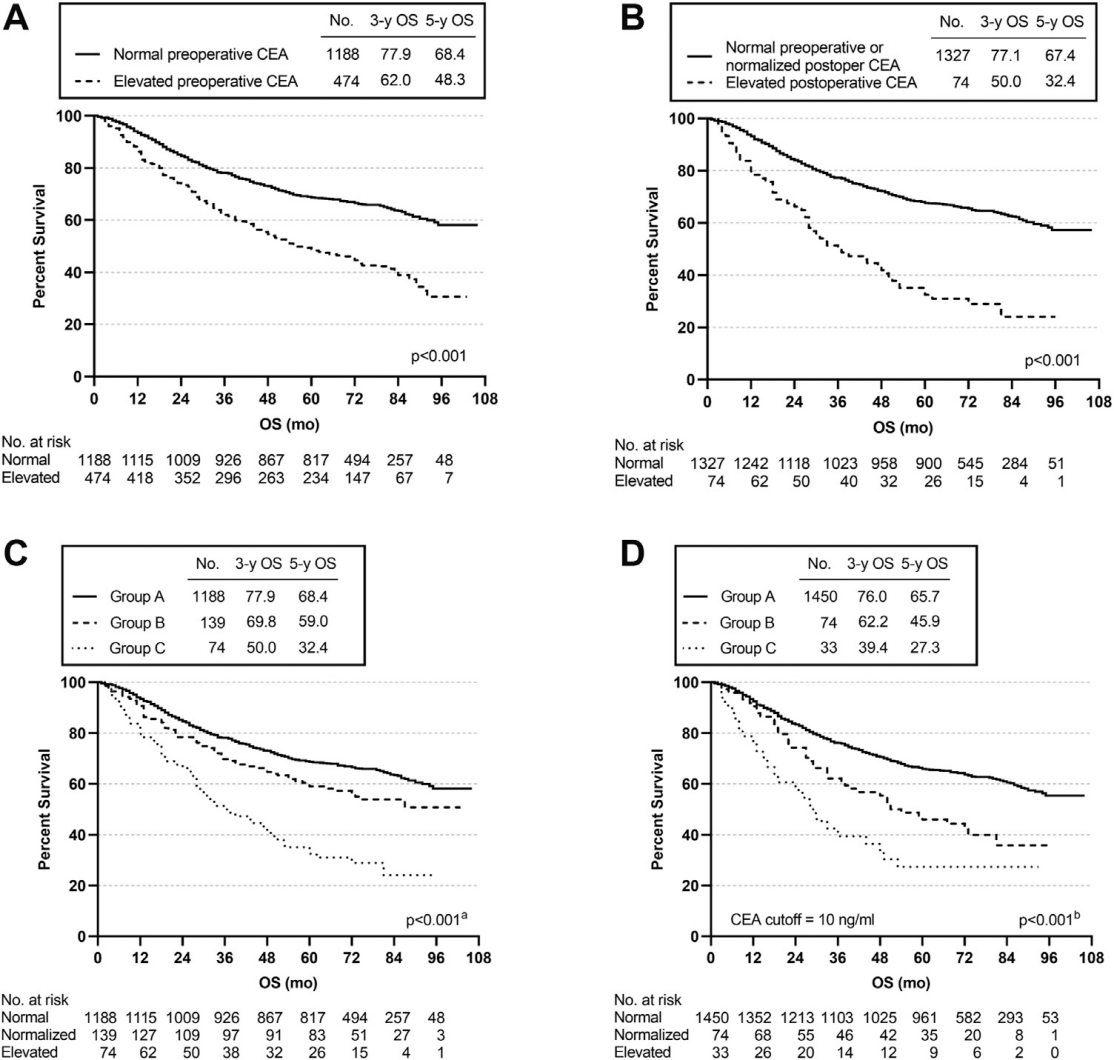
Overall survival (OS) curves according to preoperative and postoperative CEA level. **(A)** K-M curve for OS in patients with normal vs. elevated preoperative CEA. **(B)** K-M curve for OS in patients with normal preoperative or normalized postoperative CEA vs. elevated postoperative CEA. **(C)** K-M curve for OS in patients with different subgroups. **(D)** K-M curve for OS in patients with different subgroups using a CEA cut-off of 10 ng/ml. ^a^Group A vs. Group B, *p* = 0.016; Group A vs. Group C, *p* < 0.001; Group B vs. Group C, *p* < 0.001. ^b^Group A vs. Group B, *p* < 0.001; Group A vs. Group C, *p* < 0.001; Group B vs. Group C, *p* = 0.048.

Kaplan-Meier survival curves were used to assess the effect of different CEA groups. The 3- and 5-year OS rates for the 1188 patients with a normal preoperative CEA level were 77.9 and 68.4%, which were much higher than the corresponding OS rates of 62 and 48.3% for the 474 patients with an elevated preoperative CEA level (*p* < 0.001) (Figure 2A). The 3- and 5-year OS rates for the 74 patients whose CEA value remained elevated after surgery at 12 weeks were 50 and 32.4% compared with 77.1 and 67.4% for the 1327 patients with either a normalized postoperative CEA level (*n* = 139) or a normal preoperative CEA level (*n* = 1188) (*p* < 0.001) (Figure 2B). The 3- and 5-year OS rates were 69.8 and 59% for the 139 patients in group B, which were significantly different from those in group A and group C (group A vs. group B, *p* = 0.016; group A vs. group C, *p* < 0.001; group B vs. group C, *p* < 0.001) (overall log-rank *p* < 0.001) (Figure 2C). Moreover, using a CEA cut-off of 10.0 ng/ml for repeat analyses produced similar results. Group A had the best prognosis, while group C had the worst (group A vs. group B, *p* < 0.001; group A vs. group C, *p* < 0.001; group B vs. group C, *p* = 0.048) (overall log-rank *p* < 0.001) (Figure 2D).

**FIGURE 3:**
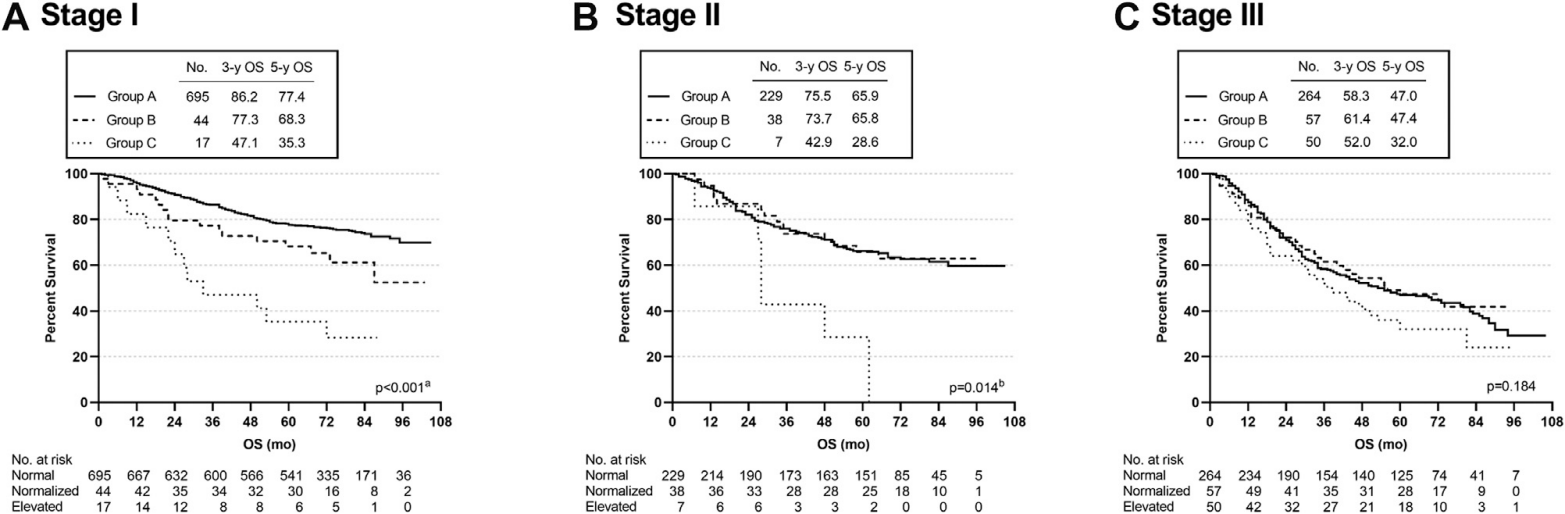
Overall survival curves of patients with different subgroups, grouped by TNM disease stage. ^a^Group A vs. Group C, *p* < 0.001; Group A vs. Group B, *p* = 0.039; Group B vs. Group C, *p* = 0.018. ^b^Group A vs. Group C, *p* = 0.004; Group B vs. Group C, *p* = 0.014.

Subgroup analyses were used to assess the effect of CEA levels on specific stages. In stage I, the OS rate was significantly different among the three cohorts (*p* < 0.001); in particular, the 3-year and 5-year OS rates of group C were observably lower than those of the other two groups (group A vs. group B, *p* = 0.039; group A vs. group C, *p* < 0.001; group B vs. group C, *p* = 0.018) (Figure 3A). In patients with stage II disease, OS was significantly lower in group C than in group A or group B (group A vs. group C, *p* = 0.004; group B vs. group C, *p* = 0.014) (overall log-rank *p* < 0.001) (Figure 3B). In stage III, the three cohorts did not differ significantly (*p* = 0.184) (Figure 3C).

**FIGURE 4:**
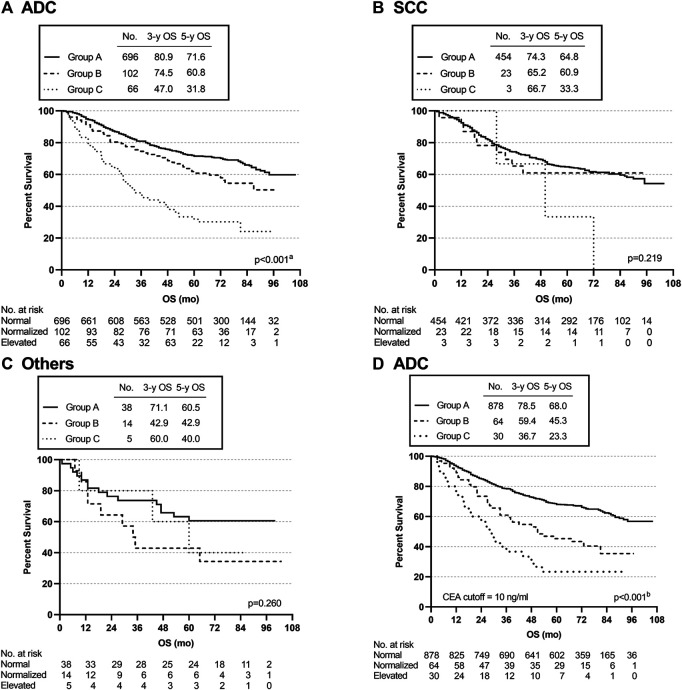
Overall survival curves of patients with different subgroups, grouped by pathological types. **(A**–**C)** K-M curves for OS in ADC, SCC, and Others. **(D)** K-M curve for OS in ADC using a CEA cut-off of 10 ng/ml. ^a^Group A vs. Group C, *p* < 0.001; Group A vs. Group B, *p* = 0.014; Group B vs. Group C, *p* < 0.001. ^b^Group A vs. Group B, *p* < 0.001; Group A vs. Group C, *p* < 0.001; Group B vs. Group C, *p* = 0.029.

Subgroup analysis was conducted according to the pathological type of lung cancer. The 3- and 5-year OS rates of the ADC patients in group B were 74.5% and 60.8, respectively, and there were statistically significant differences among the three groups (group A vs. group B, *p* = 0.014; group A vs. group C, *p* < 0.001; group B vs. group C, *p* < 0.001) (overall log-rank *p* < 0.001) (Figure 4A). However, there were no significant differences in OS between SCC and other subtypes of lung cancer (Figures 4B,C). Furthermore, using a CEA cut-off of 10.0 ng/ml for repeat analyses in ADC produced similar results (group A vs. group B, *p* < 0.001; group A vs. group C, *p* < 0.001; group B vs. group C, *p* = 0.029) (overall log-rank *p* < 0.001) (Figure 4D). Similarly, there were no statistically significant differences in OS between SCC and other pathological subtypes using a CEA cut-off of 10.0 ng/ml. The above analysis results suggested that the OS differences among the three groups (groups A, B, and C) defined by our inclusion data might be mainly reflected in lung adenocarcinoma.

**FIGURE 5:**
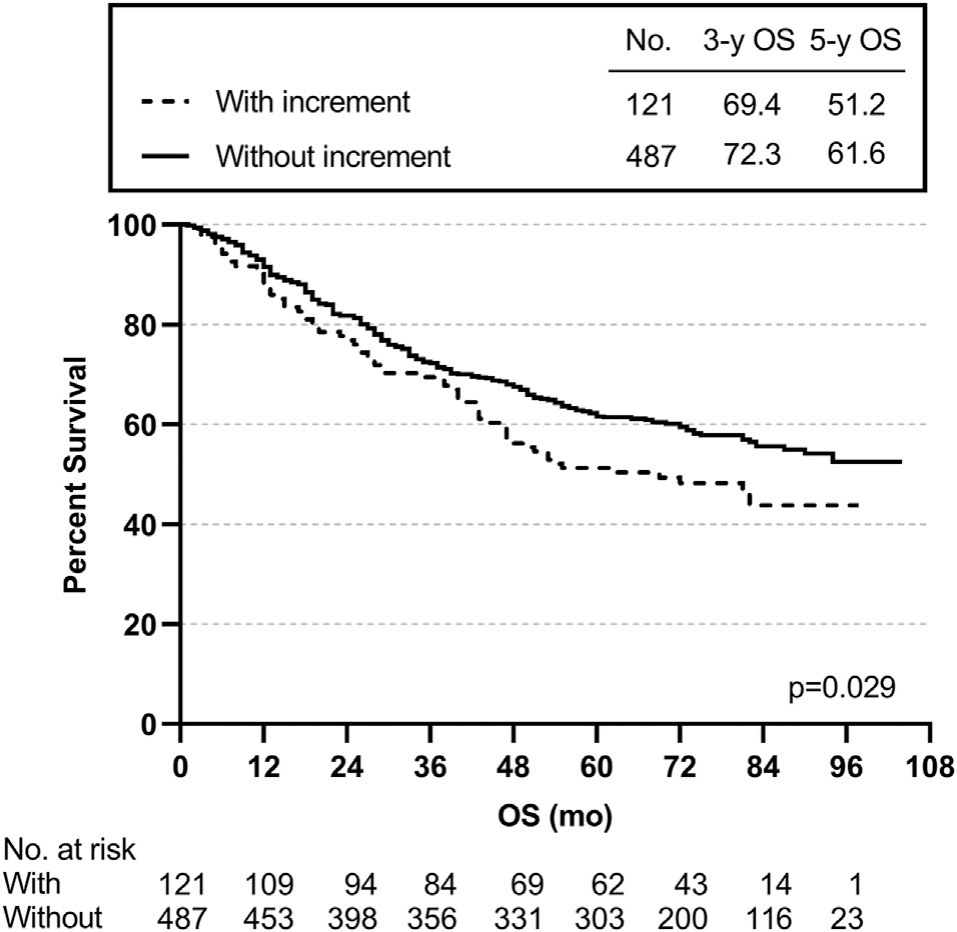
Overall survival curve by post-preoperative CEA increment.

We also created subgroups to test the effect of post-preoperative CEA on prognosis. The post-preoperative CEA increment meant that the postoperative CEA level was higher than the preoperative CEA level. The 3- and 5-year OS rates for the 121 patients with a post-preoperative CEA increment were 69.4 and 51.2% lower than the rates of 72.3 and 61.6% for the 487 patients without a post-preoperative CEA increment (*p* = 0.029) (Figure 5).

The results for univariate and multivariate analyses revealing the clinical factors associated with OS are shown in Table 3. Using K-M curves in the univariate analysis, our results suggested that sex, smoking history, TNM (tumour-node-metastasis) stage, and different groups of changes in CEA levels were associated with OS. However, age, family cancer history, and pathology had no significance for OS. Furthermore, the multivariate analyses indicated that an elevated postoperative CEA level was an independent prognostic factor for OS (HR = 2.0, 95% CI, 1.5–2.7, *p* < 0.001) rather than a normalized postoperative CEA level (HR = 1.2, 95% CI%, 0.9–1.5, *p* = 0.270). Additionally, shorter OS was associated with male sex and a higher TNM stage.

**TABLE 3:** Univariate and multivariate analyses of overall survival.

Variable	Univariate				Multivariate		
	5-year OS (%)	95% CI		*p*	Hazard ratio	95% CI	*p*
Gender				0.001			
Male	61.8	58.5–65.0			Ref		
Female	71.6	67.9–75.3			0.7	0.6–0.9	0.005
						
Age				0.285			
<65	66.4	63.7–69.1					
≥65	62.7	57.2–68.2					
						
Smoking				0.026			
No	69.6	65.9–73.3			Ref		
Yes	62.5	59.2–65.8			1.1	0.9–1.4	0.268
						
Family History				0.667			
No	65.3	62.6–68.0					
Yes	67.0	60.9–73.1					
Pathology							
				0.134			
ADC	67.2	64.1–70.3					
SCC	64.0	59.7–68.3					
Others	54.4	41.5–67.3					
						
TNM stage				<0.001			
I	75.9	72.8–79.0			Ref		
II	65.0	59.3–70.7			1.5	1.2–1.9	0.002
III	45.0	39.9–50.1			2.7	2.2–3.2	<0.001
						
CEA group				<0.001			
Normal preoperative CEA (A)	68.4	65.9–70.9			Ref		
Normalized Postoperative CEA (B)	59.0	50.8–67.2			1.2	0.9–1.5	0.270
Elevated Postoperative CEA ^©^	32.4	21.8–43.0			2.0	1.5–2.7	<0.001

In the subgroup analysis, we found that the differences in OS among the three groups might be mainly reflected in ADC. Therefore, univariate and multivariate analyses were conducted in patients with ADC. Our results indicated that sex, TNM stage, and different groups of changes in CEA levels were associated with OS. Moreover, the multivariate analyses suggested that an elevated postoperative CEA level was an independent prognostic factor for OS (HR = 1.9, 95% CI, 1.4–2.7, *p* < 0.001) rather than a normalized postoperative CEA level (HR = 1.1, 95% CI%, 0.8–1.5, *p* = 0.676). Additionally, shorter OS was closely associated with male sex and a higher TNM stage (Table 4).

**TABLE 4:** Univariate and multivariate analyses of overall survival in ADC.

Variable	Univariate				Multivariate		
	5-year OS (%)	95% CI		*p*	Hazard ratio	95% CI	*p*
Gender				0.002			
Male	62.0	57.3–66.7			Ref		
Female	72.0	67.9–76.1			0.7	0.6–0.9	0.001
						
Age				0.335			
<65	68.0	64.5–71.5					
≥65	64.4	57.3–71.5					
						
Smoking				0.183			
No	69.4	65.5–73.3					
Yes	64.1	59.2–69.0					
						
Family History				0.341			
No	66.8	63.5–70.1					
Yes	70.8	62.2–79.4					
						
TNM stage				<0.001			
I	78.1	74.6–81.6			Ref		
II	62.0	53.4–70.6			1.8	1.3–2.5	0.001
III	45.4	38.9–51.9			3.1	2.4–4.0	<0.001
						
CEA group				<0.001			
Normal preoperative CEA (A)	71.6	68.3–74.9			Ref		
Normalized Postoperative CEA (B)	60.8	51.4–70.2			1.1	0.8–1.5	0.676
Elevated Postoperative CEA (C))	31.8	20.6–43.0			1.9	1.4–2.7	<0.001

## Discussion

In our study, the data showed that changes in the perioperative CEA level provided an informative prognostic reference for patients with stage I-III lung cancer who underwent curative resection. Patients with a normal preoperative CEA level had 15.9 and 20.1% higher 3- and 5-year OS rates than those with an elevated preoperative CEA level, and this result was consistent with the literature (Icard et al., 1994; Foa et al., 1999; Suzuki et al., 1999; Okada et al., 2003; Matsuoka et al., 2007). Following surgery, more than 65% of patients had a normalized CEA level, and the outcomes of these patients were worse than those of patients with a normal preoperative CEA level but better than those of patients with an elevated postoperative CEA level. Repeat analyses using a CEA cut-off of 10.0 ng/ml obtained similar results. Furthermore, those patients with an elevated CEA level following surgery had an absolute 27.1 and 35% lower 3- and 5-year OS rates than those with either a normalized postoperative CEA level or a normal preoperative CEA level. Subgroup analyses clearly showed a significant difference in survival among the three groups in stage I, with similar trends in stages II, and revealed poor survival in patients with a post-preoperative CEA increment. However, perioperative CEA is not able to stratify patients with stage III disease, likely due to the high tumor load of stage III patients and the early recurrence and metastasis. Moreover, in our data, fewer patients were included in group B and group C, which may overestimate the survival rate of these two groups. Univariate analysis suggested that sex, smoking history, TNM stage, and different groups of changes in CEA levels were related to OS. In addition, multivariable analyses demonstrated that a persistently elevated CEA level following surgery was an independent prognostic factor for lung cancer, consistent with previous research (Wang et al., 2010; Kozu et al., 2013; Duan et al., 2015). We found that lung adenocarcinoma accounted for the majority (61.7%) of all pathological subtypes we enrolled. We conducted survival analysis according to different pathological subtypes by the K-M method and found that only lung adenocarcinoma had results similar to those reported above. Furthermore, univariate and multivariate analyses found that an elevated postoperative CEA level was an independent prognostic factor in lung adenocarcinoma. Using a CEA cut-off of 10 ng/ml, consistent trend results were obtained. This indicated that the positive results obtained in all patients enrolled in our study were probably due to the large proportion of lung adenocarcinoma patients.

The findings are similar to those of other studies (Sawabata et al., 2002; Okada et al., 2004); evaluating perioperative serum CEA levels in patients with lung cancer following surgery has significant prognostic value. In most patients, preoperative CEA levels returned to normal after surgery. These patients’ survival status was significantly better than that of patients whose postoperative CEA level remained elevated, indicating that a normalized postoperative CEA level is an essential and favourable prognostic indicator for patients with a higher preoperative CEA level than normal. We concluded that patients with persistent CEA elevation have the worst prognosis even after apparent surgical success and require more careful follow-up. Another study noted that patients with low levels of postoperative CEA (<2.5 ng/ml) had an extremely favourable prognosis compared with those with normal or elevated postoperative CEA levels among patients with pathologic stage Ia NSCLC (Sawabata et al., 2004b). However, some studies have reached the controversial conclusion that CEA is not useful as a diagnostic marker in lung cancer (Hanagiri et al., 2011; Takahashi et al., 2011). Therefore, these data must be interpreted with care. CEA may carry prognostic information, but it might not be an adequate prognostic indicator for guiding clinical decisions. The conflicting results may indicate that serum CEA alone is not sufficiently sensitive for monitoring patient outcomes; however, the combination of CT findings and pre/postoperative serum CEA levels provides reliable prognostic information for DFS and OS in lung cancer patients (Takamochi et al., 2004; Higashi et al., 2009; Yamazaki et al., 2015). In addition, the combined detection of serum tumour markers before lung cancer surgery has significant prognostic value. For example, combined detection of CEA and CYFRA21-1, tumour marker indexes, may be a promising approach for assessing patient prognosis (Muley et al., 2008; Tomita et al., 2010).

As a tumour marker with a prognostic role in lung cancer, CEA is convenient to detect during preoperative or postoperative periods and has been widely used in clinical practice. However, guidelines published by the American Thoracic Society and European Respiratory Society stated that CEA routine testing was not recommended for staging or disease prognosis (Jett et al., 1997). Furthermore, the NCCN guidelines for the management of NSCLC issued in 2021 did not recommend CEA as a pretreatment evaluation indicator (NCNN, 2020). Nevertheless, Ozeki et al. emphasized the importance of follow-up by CEA monitoring for patients after lung cancer surgery (Ozeki et al., 2014). In clinical practice, should we refer to perioperative serum CEA levels or dynamic changes in CEA levels to provide treatment or follow-up recommendations for patients with lung cancer? We believe that serum tumour markers play an important role in lung cancer, but these markers have not received enough attention in clinical practice. Therefore, our study may provide meaningful insights for clinicians. Of course, there were some limitations to our study. First, this was a retrospective study subject to the inherent limitations and biases of observational retrospective research. For example, the number of patients with a normalized postoperative CEA level or an elevated postoperative CEA level was smaller than that of patients with a normal preoperative CEA level. Second, the limitations of this observational study were typical for cohorts of patients treated at a single academic institution, including potential selection biases, generalizability, and attrition (only half of the patients with an elevated CEA level at baseline had postoperative CEA information). In comparison, prospective studies will provide more convincing evidence of the significance of CEA in lung cancer and these data need to be further verified by multiple centres. In addtion, in our study, some factors were missing or not recorded, such as comorbidities and economic status, so the suggestive effect of perioperative detection of serum CEA on the risk of death may be overestimated, and its real effect needs to be further studied. Again, we studied only one tumour marker, and the combination of multiple indicators (other tumor biomarkers or imaging methods) might provide more clinical significance. Finally, the patients in our research were all Chinese.

In conclusion, our results demonstrate the effect of dynamic changes in serum preoperative and postoperative CEA levels in a large cohort of patients undergoing resection for lung cancer. They indicate that the postoperative CEA level may inform the frequency of surveillance. Therefore, this study will provide valuable information for lung cancer patients as a clinical reference for follow-up treatment.

## Conclusion

Patients with a normalized postoperative CEA level or an elevated postoperative CEA level had worse OS than those with a normal preoperative level. Unlike a normalized postoperative CEA level, an elevated postoperative CEA level is an independent adverse prognostic factor in lung cancer patients undergoing surgery, especially in lung adenocarcinoma. It is emphasized that CEA monitoring during the perioperative period will provide more valuable prognostic information for patients with lung adenocarcinoma.

## Data Availability

The original contributions presented in the study are included in the article/Supplementary Materials, further inquiries can be directed to the corresponding author.
